# Controlled Induction of Parthenogenesis in Transgenic Rice *via* Post-translational Activation of *PsASGR-BBML*

**DOI:** 10.3389/fpls.2022.925467

**Published:** 2022-07-08

**Authors:** Gurjot Singh Sidhu, Joann A. Conner, Peggy Ozias-Akins

**Affiliations:** ^1^Institute of Plant Breeding, Genetics, and Genomics, University of Georgia, Tifton, GA, United States; ^2^Department of Horticulture, University of Georgia, Tifton, GA, United States

**Keywords:** *PsASGR-BBML*, parthenogenesis, haploid induction, glucocorticoid receptor, dexamethasone

## Abstract

Modern plant breeding programs rely heavily on the generation of homozygous lines, with the traditional process requiring the inbreeding of a heterozygous cross for five to six generations. Doubled haploid (DH) technology, a process of generating haploid plants from an initial heterozygote, followed by chromosome doubling, reduces the process to two generations. Currently established *in vitro* methods of haploid induction include androgenesis and gynogenesis, while *in vivo* methods are based on uni-parental genome elimination. Parthenogenesis, embryogenesis from unfertilized egg cells, presents another potential method of haploid induction. PsASGR-BABY BOOM-like, an AP2 transcription factor, induces parthenogenesis in a natural apomictic species, *Pennisetum squamulatum (Cenchrus squamulatus)* and *PsASGR-BBML* transgenes promote parthenogenesis in several crop plants, including rice, maize, and pearl millet. The dominant nature of *PsASGR-BBML* transgenes impedes their use in DH technology. Using a glucocorticoid-based post-translational regulation system and watering with a 100 μM DEX solution before anthesis, PsASGR-BBML can be regulated at the flowering stage to promote parthenogenesis. Conditional expression presents a novel opportunity to use parthenogenetic genes in DH production technology and to elucidate the molecular mechanism underlying parthenogenetic embryogenesis.

## Introduction

Doubled-haploid (DH) technology is used extensively in some crop breeding programs to generate homozygous lines from a heterozygous cross in two generations as compared to five to six generations with traditional breeding (Dunwell, 2010). To obtain doubled-haploids, DH technology involves inducing plants from a haploid gametophyte, followed by chromosome doubling, typically using mitotic inhibitors such as colchicine. In cross-pollinated crops such as corn and *Brassica* spp., which show a high degree of heterosis, this technology is used to develop inbred lines which are then crossed to produce superior hybrids (Ferrie and Möllers, 2011; Chaikam et al., 2019). In self-pollinated crops, such as wheat and rice, DH technology can be used to produce homozygous pure-lines from heterozygous F_1_ progeny (Weyen, 2008).

Methods to induce haploids are mainly divided into two categories—*in vitro* and *in vivo*. *In vitro* methods involve tissue-culture techniques to regenerate haploid cells from microspores or unfertilized egg cells, which are differentiated to produce embryos and ultimately haploid plants. Anther culture is a popular technique for haploid induction in crops such as rice (Mishra and Rao, 2016) but has several limitations, including genotype specificity within a species (Forster et al., 2007). Other agricultural species such as woody plants and leguminous crops are often recalcitrant to this technique (Murovec and Bohanec, 2012). The culturing of un-pollinated floral buds or isolated ovaries on tissue-culture media is a technique used for DH production in onion and sugar beet (Bohanec, 2009).

*In vivo* methods generate haploids through uni-parental genome elimination or by inducing parthenogenesis in unfertilized egg cells. In uni-parental genome elimination, the genome of one parent (inducer line) gets removed from the diploid zygote resulting from a cross between two parents. An inducer line, Stock-6, and its derivatives, have been widely used for haploid induction in maize since the advent of DH technology. Since first reported in 1959 (Coe, 1959), 60 years of intensive breeding have increased the haploid induction rate (HIR) of Stock-6 from 3% to about 15% (Uliana Trentin et al., 2020). Two major QTLs, *qhir1* and *qhir8*, respectively, explain 66 and 20% of the HIR genotypic variance (Prigge et al., 2012). *qhir1* carries a 4 bp insertion in the fourth exon of the *MATRILINEAL* (*MTL*)/*NOT LIKE DAD* (*NLD*)/*ZmPHOSPHOLIPASE A1* (*ZmPLA1*) gene, which encodes a patatin-like phospholipase expressed primarily in pollen (Gilles et al., 2017; Kelliher et al., 2017; Liu et al., 2017). The identification of *MTL*/*NLD*/*ZmPLA* provided an opportunity to engineer HI in other crops and potentially create new HI lines in maize. A *de novo MTL* mutation in a non-inducer maize line—*mtl*/*mtl* gave a maximum HIR of 12.5% and an average HIR of 6.7% (Kelliher et al., 2017). An engineered *mtl*/*mtl* rice mutant yielded an HIR of about 6% with a 20% seed-set (Yao et al., 2018), while Wang et al. (2019) observed an HIR of 3.63% with about 11.5% seed-set. Changes to wheat orthologous genes, *TaMTL-4A*, *TaMTL-4B*, and *TaMTL-4D*, and the *Setaria italica* ortholog *SiMTL*, result in HIR rates of 11.8–31.6 and 1.75–3.49%, respectively (Yao et al., 2018; Liu et al., 2019). *qhir8* was identified as a DUF679 membrane protein—ZmDMP (Zhong et al., 2019). Unlike *MTL/NLD/ZmPLA*, which is conserved across monocots, *DMP-like* genes exist both in monocots and eudicots. The identification of *ZmDMP* has allowed the engineering of HI within both monocots and dicots. Although a knock-out of wild-type *ZmDMP* resulted in a low HIR of about 0.15%, its combination with a *mtl*/*nld*/*zmpla1* mutation increased HIR by two to six-fold. This synergistic effect suggests that at least two distinct pathways play a role in Stock-6 based haploid induction (Jacquier et al., 2020). Arabidopsis mutants with loss-of-function of two *DMP-like* genes—*AtDMP8* and *AtDMP9*—have shown an average HIR of 1–3.2% (Zhong et al., 2020). Engineered *DMP* genes in *Medicago truncatula* and tomato have also been shown to produce HI (Wang et al., 2022; Zhong et al., 2022).

Uni-parental genome elimination in Arabidopsis was achieved by modifying the centromeric histone H3 (CENH3) protein, which epigenetically determines centromere location by creating chromatin onto which the kinetochore assembles (Ravi and Chan, 2010). Multiple variants of modified CENH3 have been identified that induce uni-parental genome elimination with varying efficiencies (Kuppu et al., 2020). In mature egg cells of Arabidopsis, variant CENH3 is recognized and selectively removed from centromeres creating a CENH3-depleted “weak” chromosome. During mitosis of hybrid zygotes, the CENH3-depleted centromeres do not compete as well for CENH3 and essential centromere proteins loading compared to normal centromeres leading to the missegregation/loss of weak centromeres and haploid induction (Marimuthu et al., 2021). In maize, complementing loss-of-function *cenh3*/*cenh3* mutants with a *tailswap-CENH3* resulted in an average HIR of 0.86% with a maximum HIR of 3.6% (Kelliher et al., 2016). Efforts to engineer CENH3-based haploid induction in bread wheat, an allohexaploid crop, were complicated by the presence of two *CENH3* genes (α and β). A paternal HIR rate of 7–8% was achieved by retaining six *CENH3*β alleles in wild-type state, a specific mutation in *CENH3*α*-A* (termed restored frameshift—*RFS*) coupled with knockout alleles for both *CENH3*α*-B* and *CENH3*α*-D* (Lv et al., 2020).

In parthenogenesis, the egg cell initiates embryogenesis without being fertilized by a sperm cell. Parthenogenesis in natural apomicts will retain the ploidy level of the maternal parent as the egg cells in the aposporous or diplosporous embryo sac are unrecombined and unreduced through the process of apomeiosis. Parthenogenesis in sexually reproducing diploid plants will produce meiotically recombined haploid progeny as embryogenesis will be initiated from meiotically reduced egg cells. *PsASGR-BBML*, the gene responsible for parthenogenesis in the natural apomict *P. squamulatum*, can induce parthenogenesis in sexual pearl-millet, rice, maize, and tobacco when expressed under the control of an egg cell-specific promoter (Conner et al., 2015, 2017; Zhang et al., 2020). The dominant phenotype of *PsASGR-BBML* transgenes limits its usefulness for DH technology. For the deployment of dominant parthenogenetic genes to commercial DH production pipelines, the function of PsASGR-BBML needs to be regulated such that parthenogenesis is turned on in parental lines from which haploids are desired but off in the resulting DH progeny.

The glucocorticoid receptor (GR) system has been used in plants for post-translational regulation (Yamaguchi et al., 2015). GR dependent post-translational activation systems have been successfully used to induce the downstream transcriptional responses of transcription factors such as BnBBM (Passarinho et al., 2008), LEAFY (William et al., 2004), and WUSCHEL (Leibfried et al., 2005) in Arabidopsis and OsMADS26 (Lee et al., 2008), WOX11 (Zhao et al., 2009) and WOX3 (Dai et al., 2007) in rice. A post-translational regulation system using the GR relies on a fusion construct where the gene of interest (GOI) is fused to the ligand-binding domain of rat GR. The GOI:GR fusion protein forms a cytoplasmic complex with heat shock protein 90 (HSP-90) and is retained in the cytoplasm until the application of dexamethasone (DEX), a synthetic steroid hormone. DEX binds to the GR domain and disrupts its interaction with HSP-90 which allows the GOI:GR fusion protein to relocate to the nucleus, where the GOI induces the expression of its downstream targets. Successful regulation of parthenogenesis using a post-translational activation system requires DEX mediated activation of PsASGR-BBML:GR in the egg cell before fertilization.

Using a glucocorticoid-based post-translational regulation system, we demonstrate that *PsASGR-BBML:GR* driven by an Arabidopsis egg cell-specific promoter *AtDD45* (downregulated in *dif1*) can be regulated at the flowering stage to induce a high rate of parthenogenesis. This technique presents a new opportunity to use parthenogenetic genes in DH production technology and to potentially elucidate the molecular mechanism underlying parthenogenetic embryogenesis.

## Materials and Methods

### Transformation Construct

**FIGURE 1:**
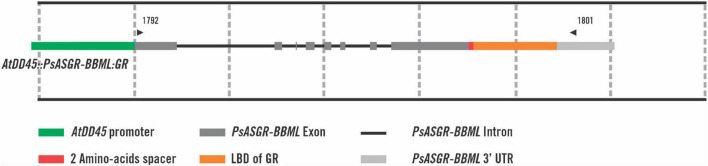
Visual representation of the *AtDD45::PsASGR-BBML:GR* transgene cassette. The green rectangle denotes the *DD45* promoter from Arabidopsis. The gray rectangles denote the *PsASGR-BBML* exons, with black lines representing introns. The red rectangle denotes a two amino acid spacer between the last amino acid of *PsASGR-BBML* and the ligand-binding domain (LBD) of GR, which is represented by the orange rectangle. The light gray rectangle denotes the native *PsASGR-BBML* 3′ UTR. Positions of primers 1792 and 1801 are represented by small black triangles.

The pCambia1300-*AtDD45::PsASGR-BBML:GR* transformation plasmid was based on the *DD45-gPsASGR-BBML* construct which was previously shown to induce parthenogenesis in rice (Conner et al., 2017). A GS-*PsASGR-BBML:GR* plasmid was produced containing a 1,089 bp synthetic DNA insert in pUC57 (GenScript USA Inc., Piscataway, NJ, United States). The GS-*PsASGR-BBML:GR* plasmid contained 125 bp of *PsASGR-BBML* exon 8 covering a unique *Bsu*36I restriction site in *DD45-gPsASGR-BBML*, a six nucleotide (two amino acid) buffer, an 852 bp GR domain sequence with one nucleotide change (T to A) to remove a *Bsu*36I restriction site from the GR domain without changing the amino acid, a TGA stop codon, and 99 bp of the *PsASGR-BBML* 3′ UTR covering a unique *Bgl*II site in *DD45-gPsASGR-BBML* (Supplementary Figure 1). The *DD45-gPsASGR-BBML* and GS-*PsASGR-BBML:GR* plasmids were digested with *Bsu*36I and *Bgl*II and the 244 bp *DD45-gPsASGR-BBML* fragment was replaced by the 1,084 bp GS-*PsASGR-BBML:GR* fragment through ligation, transformation, and verification to yield the *AtDD45::PsASGR-BBML:GR* cassette within pCambia1300 (Figure 1).

### Plant Materials

In total 22 transgenic rice lines (*Oryza sativa Nipponbare*) were generated at the CALS Plant Transformation Facility, Cornell University, after transformation with *Agrobacterium tumefaciens* possessing the *AtDD45::PsASGR-BBML:GR* cassette cloned within the *pCambia1300* binary vector. Transgenic rice plantlets were moved to soil, hardened, and grown in the NESPAL greenhouse facility, Tifton, GA, maintained at 24–29°C (Conner et al., 2017). Plants were bottom-watered with low pH water (pH 5.2–5.8) and additionally fertilized with a 50 ml per pot solution of 3.96 g/L “Miracle-Gro^®^ Water Soluble All Purpose Plant Food” and 7.8 ml/L “Ferti-Lome Chelated Liquid Iron and Other Micro Nutrients” as needed.

### Seed Sterilization and Germination

Dehusked seeds were placed in a 15 ml Falcon tube and subjected to an 80% EtOH treatment for 1 min followed by a solution of 50% commercial bleach (5.25% hypochlorite) with 0.1% Tween-20 for 30 min with rotation. The seed was washed five times with sterile water and placed on 1 × MS (Murashige and Skoog) media with vitamins (PhytoTech Labs, Lenexa, KS, United States), 4 g/L Gelzan CM (Sigma–Aldrich, St. Louis, MO, United States), 2 ml/L PPM™ (Plant Cell Technology, Washington, DC, United States) and 2% sucrose. Seeds were germinated in a 27°C incubator with a 16 h-light–8 h-dark cycle.

### DNA Extraction and Genotyping

CTAB-extracted DNA was isolated (Conner et al., 2013) and genotyped for *PsASGR-BBML:GR* using established primers 1792/1801 and PCR conditions (Conner et al., 2017).

### Transgene Expression and Cloning

Total RNA was isolated from approximately 30 rice ovaries collected 1 day before anthesis using the RNeasy Plant Mini Kit (QIAGEN, Valencia, CA, United States). One microgram of total RNA was converted into cDNA using the SuperScript^®^ III First-Strand Synthesis System kit (Invitrogen, Carlsbad, CA, United States). Expression analysis was performed using non-quantitative RT-PCR in a 25 μl PCR reaction consisting of 2 μl cDNA, 1X PrimeSTAR GXL Buffer, 200 μM dNTP, 0.2 μM p5022 (5′-AGCAAGAATAGGAAGTGTGGCA-3′)/p5023 (5′- TTGACGATGGCTTTTCCTAGCT-3′), and 0.625U PrimeSTAR GXL DNA Polymerase (Takara Bio Inc., CA, United States) for 35 cycles and an annealing temperature of 60°C. For *PsASGR-BBML:GR* transcript sequence verification, cDNA was amplified using primers 1792/1801, cloned into the Zero Blunt™ TOPO™ vector (Thermo Fisher Scientific, MA, United States), and sequenced by Psomagen, MD, United States.

### Flow Cytometry

Bulk seed flow cytometry (BSFC) using five dissected embryos from mature seed for one sample, embryos from a single seed, and leaf flow cytometry were done according to Conner et al. (2017) with *Paspalum notatum* as the genome standard to determine ploidy level. Flow cytometry and ploidy level plots were generated using a BD Accuri C6 flow cytometer and software (BD Biosciences, San Jose, CA, United States). Background signal was removed through gating of signals that exhibited a strong correlation between FL2-A and FSC-A plotted on a logarithmic scale. Ploidy level plots are based on a linear Y-axis count number and a logarithmic X-axis FL2-A signal.

### Seed Data

Three to four panicles collected from a study sample were randomly picked. The number of total mature seeds divided by the number of florets on the panicles multiplied by 100 gave seed set percentage data. The seed count was determined by the collection of all mature seeds from sample panicles. A fifty-seed weight was obtained for each plant sample. The weight of the total seed sample was measured and the seed count was calculated by [(total seed weight)/(50 seed weight)] × 50.

### Whole Plant Dexamethasone Treatments

A 100 mM stock solution of dexamethasone (Sigma-Aldrich) was prepared in dimethyl sulfoxide (DMSO) (Sigma-Aldrich) and stored in aliquots at −20°C. For the initial DEX study, *PsASGR-BBML:GR* transgene positive plants were treated with 20, 50, or 100 μM DEX solution for 10–14 days, beginning at the boot stage for the first panicle of the plant sample and ending at the time when the first flush of flowering was complete. Flowering panicles were tagged. DEX working solutions were prepared by diluting the stock solution in low pH water (pH 5.2–5.8). The containers were watered with the minimum amount of DEX solution used daily by the rice plants, such that the containers were not dry but had a minimal amount of solution left. A fresh solution was added to the remaining solution daily. Transgene positive plants treated with 0.05 and 0.1% DMSO, and wild-type rice treated with all three concentrations of DEX and 0.05 and 0.1% DMSO were used as controls. For the second DEX study, plant treatment was started when the first plant in the treatment trial reached the boot stage and continued for 14 days. The treatments included 100 μM DEX, 0.1% DMSO, and only water.

### *In planta* Floral Dexamethasone Treatment

#### Emasculated Florets

Florets from one panicle of a given T_1_ plant were emasculated approximately 1 day before the first floral opening and ovaries were bathed with 5 μl of 100 μM DEX dispensed by pipette for three consecutive days. Florets from another panicle of the same plant were emasculated and treated with 0.1% DMSO. Ovaries were collected 5 days after emasculation and fixed overnight in FAA (47.5% ethanol, 3.7% formaldehyde, and 5% acetic acid). Fixed ovaries were dehydrated successively in 70, 85, and 100% ethanol for 2 h and transferred to 100% ethanol overnight. Dehydrated ovaries were cleared successively in 2:1 ethanol:methyl salicylate (MS), 1:2 ethanol:MS, and pure MS for 2 h for each treatment and then transferred to pure MS overnight. Cleared ovaries were observed under differential interference contrast (DIC) microscopy.

#### Un-Emasculated Florets

Floret tops from one panicle of a given T_1_ plant were cut without disturbing the anthers and treated with 5 μl of 100 μM DEX for three consecutive days. Floret tops from another panicle from the same plant were cut in a similar way followed by 0.1% DMSO treatment. Upon maturity, seeds were collected and analyzed by BSFC.

## Results

In total, 22 independent *AtDD45::PsASGR-BBML:GR* lines, some with multiple plantlets, were generated and transferred to soil. Twenty-one lines had at least one *AtDD45::PsASGR-BBML:GR* transgene positive plant as determined by PCR with primers 1792/1801 (Figure 1). Expression of *PsASGR-BBML:GR* in rice ovaries 1 day before anthesis was confirmed by RT-PCR analysis from 10 independent lines. *PsASGR-BBML:GR* transcript sequence and splicing were verified from two lines. BSFC on 20 T_1_ seed from DEX-untreated *PsASGR-BBML:GR* expressing T_0_ lines did not show haploid embryo signal, verifying that in the absence of DEX, PsASGR-BBML:GR did not localize to the nucleus and parthenogenesis was not induced. Line 13B was not analyzed by BSFC due to a low seed set (Table 1).

**TABLE 1:** Characterization of *AtDD45::PsASGR-BBML:GR* T_0_ lines used in DEX induction studies.

Line	Transgene	Transcript expression *via* RT-PCR analysis	BSFC of T_1_ seed	T_1_ seeds germinated/total sown	Transgene positive T_1_/germinated
4C	+	Y	2n	12/15	7/12
6B	+	Y	2n	6/10	4/6
7B	+	Y	2n	7/10	5/7
8B	+	Y	2n	7/10	7/7
9B	+	Y	2n	8/10	7/8
10B	+	Y	2n	9/15	8/9
11B	+	Y	2n	10/10	7/10
13B	+	Y	Low seed	8/10	7/8
15B	+	Y	2n	8/15	7/8
22B	+	Y	2n	8/15	7/8

### Dexamethasone Treatment of T_1_ Plants

**FIGURE 2:**
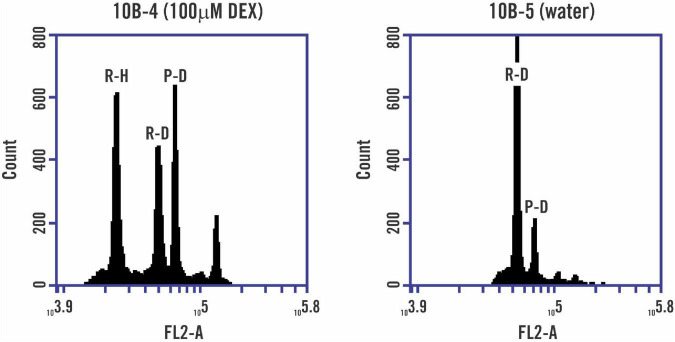
Representative BSFC to identify parthenogenesis. Embryos from five seeds were bulked, processed, and analyzed with flow cytometry to determine if PsASGR-BBML:GR would induce haploid embryo development when induced with DEX. Line 10B-5, transgene positive, without DEX treatment did not display a haploid signal. 10B-4, transgene positive, with 100 μM DEX treatment displayed both haploid and diploid rice signals from bulked embryo tissue. R-H (rice haploid signal), R-D (rice diploid signal), and P-D (*Paspalum notatum* diploid signal). The plots shown are based on a linear Y-axis count number and a logarithmic X-axis FL2-A signal.

Germination and transgene genotyping results are shown for T_1_ seed from 10 T_0_ lines (Table 1). A total of 66 out of 83 T_1_ plants were genotyped as transgene positive, and all T_1_ plants were confirmed to be diploid following flow cytometry on T_1_ leaf tissue. Expression of *PsASGR-BBML:GR* in ovaries 1 day before anthesis was confirmed by RT-PCR for 13 T_1_ plants from seven lines. One transgene positive T_1_ plant from each T_0_ line and two wild-type (WT) plants were treated with 100 μM, 50 μM, or 20 μM DEX through bottom watering (Supplementary Figure 2). Transgene positive plants treated with DMSO, WT plants treated with all three DEX concentrations, and untreated transgene positive plants were used as controls. Treatment began at the boot stage and continued for 10–14 days, encompassing the first flush of panicle flowering. Panicles from the treatment period were flagged and the seed was allowed to mature. Data for haploid embryo development was collected using BSFC on mature seed embryos and the percent seed set of flagged panicles is presented (Table 2 and Figure 2).

**TABLE 2:** BSFC results and seed set from DEX treatment through bottom watering of T_1_ transgene positive plants.

100 μM DEX			50 μM DEX			20 μM DEX			DMSO			Untreated		
T_1_ plant ID	BSFC^#^	Seed set	T_1_ plant ID	BSFC^#^	Seed set	T_1_ plant ID	BSFC^#^	Seed set	T_1_ plant ID	BSFC^#^	Seed set	T_1_ plant ID	BSFC^#^	Seed set
4C-1	3/4	32.7%	4C-5	4/4	34.9%	4C-7	0/5	51.9%						
6B-2	4/4	26.8%	6B-3	n/a	0.0%	6B-1	n/a	0.0%				6B-6	0/3	11.0%
7B-5	0/4	37.7%	7B-2	n/a	0.0%	7B-1	0/3	19.6%						
8B-3	0/4	23.6%	8B-2	0/4	18.8%	8B-1	0/5	51.1%	8B-4^∧^	0/5	39.5%			
9B-3	4/4	45.0%	9B-2	0/3	13.9%	9B-1	1/5	53.5%	9B-4*	0/5	7.6%	9B-6	0/4	77.4%
10B-4	4/4	27.5%	10B-3	3/4	28.1%	10B-1	3/5	43.5%				10B-5	0/4	54.4%
11B-3	0/4	43.8%	11B-2	0/4	50.5%	11B-1	0/5	93.1%						
13B-3	3/4	45.5%	13B-2	n/a	0.0%	13B-1	n/a	0.0%	13B-5*	0/5	43.8%	13B-6	0/4	74.5%
15B-4	3/4	30.7%	15B-2	4/4	36.9%	15B-1	0/5	41.8%	15B-3^∧^	0/4	45.9%			
22B-3	4/4	18.7%	22B-2	4/4	25.7%	22B-1	0/1	12.0%				22B-4	0/4	42.7%
WT-1	0/4	92.7%	WT-3	0/4	78.6%	WT-5	0/4	91.7%	WT-7*	0/4	96.8%			
WT-2	0/4	88.5%	WT-4	0/4	59.1%	WT-6	0/4	95.0%	WT-8*	0/4	95.0%			
									WT-9^∧^	0/4	98.8%			
									WT-10^∧^	0/4	91.8%			

*^#^Number of BSFC samples (5 embryos/sample) showing haploid signal/total number of samples.*

*Treatment with 0.05% DMSO/∧treatment with 0.1% DMSO.

In total, seven of 10 transgenic lines exhibited T_2_ seed showing haploid signal in BSFC when treated with the 100 μM DEX through bottom watering. Four lines showed haploid embryo formation at 50 μM DEX and two lines displayed haploid formation at 20 μM DEX treatment through bottom watering. Wild-type plants treated with DEX or DMSO, transgene positive plants treated only with DMSO or untreated WT and transgene positive plants did not display haploid signal in BSFC, indicating that both the *AtDD45::PsASGR-BBML:GR* transgene and inducer steroid DEX are required for successful induction of parthenogenesis. Wild-type plants treated with DMSO had an average and median seed-set of 95.6 and 95.9%, respectively, while DEX treated plants had an average and median seed-set of 84.3 and 90.1%, respectively. The overall seed set of transgene positive lines was reduced compared to WT plants and variable within T_1_ plants within and between lines.

### Identification of Transgene Homozygous Lines

From the initial 10 T_0_ lines used for DEX treatment through bottom watering, lines 4C, 10B, 15B, and 22B were selected for further analysis as BSFC haploid signals in these lines were identified in T_2_ seed when treated with 100 μM as well as 50 μM DEX through bottom watering. In total, thirty T_2_ seeds were germinated and genotyped from four T_1_ plants from each of the four lines. Six T_1_ plants (4C-14, 10B-5, 10B-11, 10B-14, 15B-3, and 22B-13) generated only transgene positive T_2_ plants and were considered homozygous for the transgene. A proportion of seedlings within 15B-3 and other 15B lines displayed an albino phenotype and were removed from further study. Of the transgene homozygous lines identified, T_1_ line 10B-14 had been treated with 100 μM DEX through bottom watering, while T_1_ line 10B-5 was an untreated bottom watering control. All 29 10B-5 offspring were diploid, while three 10B-14 seedlings were diploid and 20 seedlings were haploid.

**FIGURE 3:**
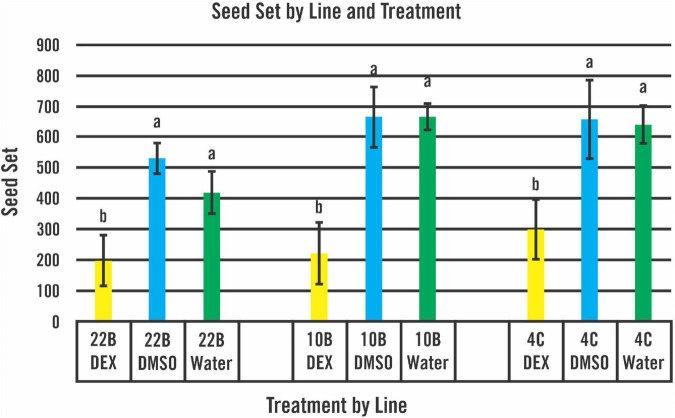
Seed set of homozygous T_2_ lines by treatment and line. A mature seed number was calculated for the first 10 flowering panicles. The number of plants treated for each line and treatment is found in Supplementary Table 1. Plants in all three lines treated with 100 μM DEX displayed significantly less mature seeds than plants treated with DMSO or no treatment controls. Error bars based on SD. Mean totals for seed set within a line not sharing a common letter were significantly different (*P* < 0.01) as determined by ANOVA using the Holm-Sidak comparison method.

New T_2_ seedlings from homozygous lines 10B-11, 22B-13, and 4C-14, confirmed to be 100% transgene positive, were placed through another round of 100 μM DEX through bottom watering with untreated and DMSO controls. Seeds from the first 10 flowering panicles were collected and the seed number was calculated based on a 50 seed weight from each plant analyzed (Supplementary Table 1). T_2_ lines from 10B-11 and 4C-14 showed more seed set from the three treatments than line 22B-13. As shown in Figure 3, all lines treated with 100 μM DEX showed significantly less seed set than plants within lines that were untreated or treated with DMSO only.

T_3_ seeds were sterilized and germinated on MS media from three T_2_ plants on three different days in DEX treatment before anthesis. The ploidy level of each germinated seedling was investigated by leaf flow cytometry. When an individual seed produced twin seedlings, each seedling was analyzed separately for ploidy level (Table 3). T_2_ lines from 10B-11 showed the greatest average level of parthenogenesis (48%), followed by T_2_ lines from 22B-13 (34%) and 4C-14 (22%). The rate of seed germination from heads decreased in all three lines the longer the plant was in DEX treatment before anthesis. While the rate of parthenogenesis and the number of twin haploid seedlings increased in lines from 10B-11 with the amount of time in DEX prior to anthesis, a similar trend was not identified in lines from 22B-13 or 4C-14.

**TABLE 3:** Ploidy level of T_3_ seedlings from homozygous T_2_ lines treated with 100 μM DEX.

Plant	Days in DEX prior to anthesis	Seed number on MS plates	Percent germination	Parthenogenesis		Sexual reproduction		Percent parthenogenesis
				Number of seed producing one haploid seedling	Number seed producing twin haploid seedlings	Number of seed producing one diploid seedlings	Number of seed producing twin diploid and haploid seedlings	
10B-11-41	4	32	91	10	2	14	3	41
10B-11-31	6	32	75	9	0	12	2	39
10B-11-39	9	32	59	3	10	2	4	68
Total 10B-11				34		37		48
22B-13-33	6	36	78	1	17	5	5	21
22B-13-32	9	34	56	3	4	3	9	63
22B-13-31	11	36	58	3	11	5	2	24
Total 22B-13				23		45		34
4C-14-31	5	34	94	1	3	23	5	12.5
4C-14-33	7	33	76	3	5	15	2	32
4C-14-34	9	35	60	2	3	11	4	25
Total 4C-14				17		60		22

### Additional Technique to Induce Parthenogenesis

An *in planta* floral treatment was attempted on transgene positive T_1_ plants 4C-11 and 4C-13, 10B-11 and 10B-13, and 22B-13. A total of 291 and 104 florets were treated with 100 μM DEX or 0.1% DMSO, respectively, and allowed to mature. Forty-five (13.4%) 100 μM DEX treated and 21 (20%) 0.1% DMSO treated florets produced seed, while an average of 81% seed set was observed on untreated florets. Seeds developed from 100 μM DEX treated florets of T_1_ plants from the 10B line displayed haploid signal with BSFC while 0.1% DMSO and untreated seeds gave diploid signal only. In total, twenty-eight florets from the 10B-11 line were emasculated, treated with 100 μM DEX for 3 days, and fixed on the fifth day after emasculation. Seventeen ovaries showed clear parthenogenesis, i.e., the appearance of a parthenogenetic embryo along with the presence of intact polar nuclei (Supplementary Figure 2).

## Discussion

Doubled haploid technology has proven to be a valuable asset to modern hybrid breeding programs due to its ability to generate inbred lines in a considerably shorter period of time as compared to traditional breeding methods. Understanding the genetic principles of haploid induction, along with the ability to modify plants through transformation and CRISPR/Cas9 technology has allowed the genetic engineering of haploid induction in various crop plants. With the identification of parthenogenesis genes, a new haploid induction technology is possible that involves direct embryogenesis from unfertilized egg cells rather than uni-parental genome elimination after crossing. Parthenogenetic haploid induction in transgenic plants has been shown by using *PsASGR-BBML* transgenes, cloned from the apomixis locus of *P. squamulatum* (syn. *C. squamulatus*), in pearl millet, rice, maize, and tobacco (Conner et al., 2015, 2017; Zhang et al., 2020) and by egg-cell specific expression of the endogenous *OsBBM1* gene from rice (Khanday et al., 2019). A dicot apomixis locus parthenogenesis gene (*PARTHENOGENESIS [PAR]*) from *Taraxacum* has been identified but has yet to produce viable haploid seedlings in transgenic lettuce lines. Parthenogenesis has been confirmed through a haploid signal from developing seeds through flow cytometry and imaging of embryo-like structures in unpollinated ovaries (Underwood et al., 2022).

Regulating PsASGR-BBML function using a GR ligand-dependent post-translational regulation system, depends both on a functional PsASGR-BBML:GR fusion protein as well as the availability of the activating ligand occurring in the desired spatial and temporal frame. The PsASGR-BBML:GR fusion protein was expressed in rice egg cells using the Arabidopsis egg cell-specific promoter *AtDD45* (Steffen et al., 2007) which has been shown to be functional to produce haploid rice lines (Conner et al., 2017; Khanday et al., 2019). Lines displaying *PsASGR-BBML:GR* expression were identified through RT-PCR on RNA extracted from pre-anthesis rice ovaries. Studies of fusion proteins with GR ligand-dependent post-translational regulation within rice (Dai et al., 2007; Lee et al., 2008; Zhao et al., 2009) and GR dependent transcriptional activation in rice (Ouwerkerk et al., 2001) have been published. The rice studies used a range of DEX concentrations from 1 to 100 μM. This study shows that GR ligand-dependent post-translational regulation within gametophytic tissue can be accomplished in rice through bottom watering of plants with the chemical ligand solution. Various lines of *AtDD45::PsASGR-BBML:GR* watered with various DEX concentrations starting at the boot stage successfully induced parthenogenesis. The number of lines showing parthenogenesis was greatest at the 100 μM DEX treatment (70%) and decreased at the 50 μM (57%) and 20 μM (25%) DEX concentrations. DEX treatment through bottom watering is an easy method and its simplicity indicates that it could have a high potential for use in large-scale commercial DH development programs. Parthenogenesis could also be induced through an *in planta* floral treatment, which is a more direct placement of the DEX solution into the rice floret. This treatment consumes additional time and limits the seed set but greatly decreases the amount of DEX solution needed for the study.

The initial DEX through bottom watering treatment indicated that neither DEX nor DMSO treatment of wild-type plants greatly affected the seed set. However, *AtDD45::PsASGR-BBML:GR* lines treated with DEX tended to show less fecundity. This result is similar to decreased fecundity levels identified in rice and maize plants expressing *AtDD45-gPsASGR-BBML* or *gPsASGR-BBML* transgenes (Conner et al., 2017). Homozygous *AtDD45::PsASGR-BBML:GR* T_1_ lines were identified and a second DEX through bottom watering study was initiated. As DEX concentration did not seem to affect WT plants, the 100 μM DEX concentration was chosen. Induction of parthenogenesis *via* DEX in homozygous T_2_ lines shows a statistical decrease in seed set for the three transgenic lines when compared to DMSO treated or untreated plants in the line, which had similar amounts of seed set. While only based on a small study, the percent germination of seed in all 3 homozygous lines declined the longer the plant was treated with DEX before the first day of anthesis for that panicle. Similar to results from *AtDD45-gPsASGR-BBML* or *gPsASGR-BBML* transgenic lines in rice, the number of twin haploid seedlings found could be greater than the number of single haploid seedlings. It is yet to be determined if the *PsASGR-BBML* transgenes promote twinning of a developing zygote or if the leaky expression of the *PsASGR-BBML* transgenes converts synergid cells into egg/zygote cell fates. The identification of a seed containing both a diploid and a haploid seedling suggests the latter. The overall haploid induction rate for homozygous T_2_ lines from 10B-11 was 48%, 34% for lines from 22B-13, and 22% for lines from 4C-14. The HI rate for these lines exceeds that of *mtl* engineered rice lines (4–6%) and is similar to or better than the dominant haploid induction rate of *PsASGR-BBML* or *OsBBM1* transgenes (Conner et al., 2017; Khanday et al., 2019). Additional studies can be undertaken to optimize the DEX treatment for concentration, timing, and testing of additional DEX-like steroids (Samalova et al., 2019).

Understanding the molecular pathways involved in PsASGR-BBML-induced parthenogenesis can also provide more options for improving the system’s efficiency for both DH production and apomixis synthesis in crop plants. Although apomixis has been synthetically engineered in rice using different approaches, the understanding of the underlying molecular mechanisms is still limited (Khanday and Sundaresan, 2021). FIX (Fixation of hybrids) rice lines were engineered with CRISPR/Cas9 technology to create a quadruple gene mutation of *osd1/pair1/rec8/mtl* (Wang et al., 2019). The phenotype of *osd1/pair1/rec8* is designated *MiMe* (*Mitosis instead of Meiosis*) and creates both male and female clonal diploid gametes (d’Erfurth et al., 2009; Mieulet et al., 2016). The *mtl* phenotype creates embryo development of the diploid egg cell, leading to clonal seed. *S-Apo* (*Synthetic-Apomictic*) rice lines were engineered using CRISPR/Cas9 technology to create a *MiMe* phenotype along with an *OsBBM1* transgene promoting embryo development of the egg cell *via* parthenogenesis (Khanday et al., 2019). The frequency of apomictic/clonal seeds ranged from 0.2 to 0.4% in the FIX lines and 14–29% in the *S-Apo* lines.

The successful post-translational regulation of PsASGR-BBML reported in this study could be used for identifying downstream target genes by using differential expression analysis in DEX treated vs. untreated *AtDD45::PsASGR-BBML:GR* plants (Yamaguchi et al., 2015). Direct transcriptional targets of PsASGR-BBML can be identified by the simultaneous application of cycloheximide, an inhibitor of protein biosynthesis. This approach has been used to identify candidate genes activated by BBM:GR during somatic embryogenesis in Arabidopsis (Passarinho et al., 2008). Chromatin immunoprecipitation (ChIP) coupled with quantitative PCR or ChIP-seq could investigate the transcriptional targets of PsASGR-BBML, similar to a study of BBM in Arabidopsis using BBM: YFP/BBM-GFP instead of GR (Horstman et al., 2017). Protein pull-down using a GR-specific antibody could also determine if PsASGR-BBML is part of a larger protein complex. A recent study used a combination of glucocorticoid-based post-transcriptional regulation and ChIP-seq to determine that *OsBBM1* induces somatic embryogenesis by directly upregulating auxin-biosynthesis genes (Khanday et al., 2020).

The data presented shows that a glucocorticoid-based post-translational regulation system using a *PsASGR-BBML:GR* transgene can be activated at the flowering stage to promote a high rate of parthenogenesis and represents a novel opportunity to use parthenogenesis genes in DH production technology.

## Data Availability Statement

The original contributions presented in this study are included in the article/Supplementary Material, further inquiries can be directed to the corresponding author.

## Author Contributions

PO-A and JAC conceptualized the project. GS and JAC did the methodology, investigation, and analysis. JAC supervised the project. GS wrote the original draft. All authors revised and approved the manuscript.

## Conflict of Interest

The authors declare that the research was conducted in the absence of any commercial or financial relationships that could be construed as a potential conflict of interest.

## Publisher’s Note

All claims expressed in this article are solely those of the authors and do not necessarily represent those of their affiliated organizations, or those of the publisher, the editors and the reviewers. Any product that may be evaluated in this article, or claim that may be made by its manufacturer, is not guaranteed or endorsed by the publisher.

## Supplementary Material

Supplementary Figure 1 Annotated GS-*PsASGR-BBML:GR* insert sequence.

Supplementary Figure 2 DEX treatment and DIC screening for parthenogenesis.

Supplementary Table 1 Individual plant data generated for second DEX study.
